# Impact of the type of glucose monitoring on the assessment of glycemic variability in critical care patients

**DOI:** 10.1186/cc10776

**Published:** 2012-03-20

**Authors:** P Kalfon, M Chilles

**Affiliations:** 1 CH Chartres, France

## Introduction

While minimizing glycemic variability (GV) during glucose control in critical care patients could become a new therapeutic goal, it is important to have a reliable assessment of GV. The aim of the study is to compare a real continuous glucose monitoring (CGM) system in comparison with a semi-continuous glucose monitoring (sCGM) system, with respect to the reliability of a marker of GV.

## Methods

We used the Eirus™ system (DipylonMedical, Solna, Sweden) based on microdialysis for CGM in three mechanically ventilated patients necessitating continuous intravenous insulin. The CGM system consists of a dedicated triple-lumen central venous catheter, a disposable sensor on a reusable sensor holder outside the patient, and a monitor producing a new measurement each minute. Calibrations were performed every 8 hours using arterial blood samples and a blood gas analyzer (Radiometer SAS, Neuilly-Plaisance, France). The attending nurses also performed intermittent blood glucose measurements with a point-of-care glucometer in order to set the insulin rate according to our glucose control method. GV was assessed by the variability index defined as the mean of the absolute value of the first derivative of the glucose signal during CGM. In order to simulate sCGM, we extracted CGM values every 15 minutes for calculating the corresponding variability index as the mean of the absolute value of the variation rate between two consecutive measurements.

## Results

The variability indexes were respectively 1.97, 1.65, 1.55 mmol/l/hour for CGMS, and 1.07, 0.65, 0.83 mmol/l/hour for sCGMS.

## Conclusion

sCGM in comparison with CGM may considerably underestimate a marker of GV during glucose control in critical care patients.
